# 分散膜萃取-超高效液相色谱-串联质谱法测定海水中8种全氟及多氟烷基化合物

**DOI:** 10.3724/SP.J.1123.2025.04009

**Published:** 2025-11-08

**Authors:** Zhen REN, Jiping MA, Yajing LIU, Lan ZHANG, Gege WU, Shuang LI

**Affiliations:** 1.青岛理工大学环境与市政工程学院，山东 青岛 266520; 1. School of Environmental and Municipal Engineering，Qingdao University of Technology，Qingdao 266520，China; 2.山东省青岛生态环境监测中心，山东 青岛 266003; 2. Qingdao Ecological Environment Monitoring Center of Shandong Province，Qingdao 266003，China; 3.日照市质量检验检测研究院，山东 日照 276800; 3. Rizhao Quality Inspection and Testing Research Institute，Rizhao 276800，China

**Keywords:** 全氟及多氟烷基化合物, 分散膜萃取, 阳离子型金属有机骨架, 超高效液相色谱-串联质谱法, per- and polyfluoroalkyl substances （PFASs）, dispersive membrane extraction （DME）, cationic metal-organic framework, ultra-high performance liquid chromatography-tandem mass spectrometry （UHPLC-MS/MS）

## Abstract

基于阳离子型金属有机骨架膜材料，建立了一种分散膜萃取（DME）与超高效液相色谱-串联质谱（UHPLC-MS/MS）相结合的分析方法，用于同时检测海水中8种全氟及多氟烷基化合物（PFASs）。优化的色谱-质谱条件如下：ACQUITY UPLC BEH C18色谱柱（100 mm×2.1 mm， 1.7 μm），进样体积为10 μL，柱温保持在40 ℃，流速为0.4 mL/min，采用1.0 mmol/L乙酸铵水溶液和乙腈为流动相进行梯度洗脱；在负离子模式下，通过电喷雾离子源（离子源电压-2 500 V，离子源温度300 ℃）进行质谱检测，并使用多反应监测模式采集化合物质谱信息。在最优条件下，8种PFASs在各自的浓度范围内线性关系良好，相关系数均≥0.990 7，方法的检出限为0.07~0.49 ng/L，定量限为0.22~1.63 ng/L。在10、50和100 ng/L加标水平下PFASs的回收率为50.4%~116.4%，日内和日间相对标准偏差分别为1.0%~19.2%和2.2%~19.5%。将该方法应用于胶州湾表层海水中8种PFASs的检测，共检出7种PFASs。其中，全氟-11-氯-3-氧杂十一烷磺酸检出浓度最高，平均质量浓度为17.11 ng/L。与2018年胶州湾表层海水中PFASs的检出结果对比，全氟辛酸的平均质量浓度水平明显降低。同时新型PFASs中全氟-9-氯-3-氧杂壬烷磺酸钾在胶州湾表层海水中被检出，可能与近些年PFASs的生产转型有关。新型PFASs的广泛使用可能带来与传统PFASs类似的环境风险，需引起人们高度关注。综上所述，本方法操作简便、快速且灵敏度高，适用于海水中8种PFASs的分析。

## Abstract (en)

Per- and polyfluoroalkyl substances （PFASs） are widely utilized in various industrial applications. Their persistence in ecosystems raises significant global environmental health concerns. Numerous studies have confirmed the toxicity of PFASs to human endocrine and immune systems. The carcinogenic risks associated with PFASs exposure increasingly alarm public health authorities worldwide. As a result， regulatory policies have been implemented to restrict both the production and environmental release of PFASs. In China， the GB 5749-2022 standard establishes stringent limits for PFASs concentrations in drinking water， capping levels of perfluorooctane sulfonic acid （PFOS） at 40 ng/L and perfluorooctanoic acid （PFOA） at 80 ng/L. Effective monitoring of PFASs requires advanced analytical techniques that exhibit exceptional sensitivity. Ultra-high performance liquid chromatography-tandem mass spectrometry （UHPLC-MS/MS） has emerged as the preferred technique for detecting these substances due to its superior selectivity and low detection limits. However， complex environmental matrices necessitate optimized sample pretreatment strategies to enhance analysis accuracy. Efficient extraction methods must effectively address matrix interference while ensuring analyte enrichment. Dispersive membrane extraction （DME） presents distinct advantages for the pretreatment of PFASs； its operational simplicity is complemented by high enrichment capabilities and efficient mass transfer. The selection of extraction materials critically influences DME performance parameters. Metal-organic frameworks （MOFs） are porous materials composed of organic ligands and metal clusters， featuring tunable pores， high surface areas， and rapid mass transfer. These unique properties enable their current use in sample pretreatment for environmental analysis. This study integrates DME with UHPLC-MS/MS utilizing cationic MOF membranes， enabling simultaneous detection of eight different PFASs within seawater matrices. Optimized parameters ensure reliable quantification of trace-level contaminants throughout this process. The experimental design assessed various parameters， including types of organic solvents （methanol and acetonitrile）， ammonium acetate concentrations in the aqueous phase （0.1， 0.5， 1.0， 2.0， and 5.0 mmol/L）， as well as ion source voltages （-2 500， -3 500， and -4 500 V）， all of which influenced detection efficacy. Chromatographic separation was performed using an ACQUITY UPLC BEH C18 column （100 mm×2.1 mm， 1.7 μm）. Gradient elution combined 1 mmol/L ammonium acetate with acetonitrile for optimal results. Detection was conducted utilizing electrospray ionization （ESI） in negative ion scanning mode coupled with multiple reaction monitoring （MRM）. The results indicated that all eight PFASs could be effectively separated within a timeframe of 12 min， exhibiting favorable peak shapes and high response values. Under optimal conditions， the eight PFASs demonstrated strong linearity across their respective concentration ranges； the correlation coefficients （*r*²） were all not less than 0.990 7. Recoveries of PFASs at spiking levels of 10， 50， and 100 ng/L ranged from 50.4% to 116.4%， while intra-day and inter-day relative standard deviations （RSDs） varied from 1.0% to 19.2% and from 2.2% to 19.5%， respectively. The method’s limits of detection （LODs） ranged from approximately 0.07 ng/L to 0.49 ng/L， whereas the limits of quantification （LOQs） varied from around 0.22 ng/L to 1.63 ng/L. Jiaozhou Bay serves as a typical semi-enclosed bay along the Pacific coast where terrestrial runoff converges nutrients alongside diverse organic/inorganic pollutants that pose potential health risks for aquatic organisms inhabiting these waters. This method was utilized for the detection of the eight PFASs in the surface seawater of Jiaozhou Bay， and seven types were successfully identified. Among these， potassium 11-chloroeicosafluoro-3-oxaundecane-1-sulfonate （Minor F-53B） exhibited the highest detection concentration， with an average mass concentration of 17.11 ng/L. When compared to the 2018 detection results of PFASs in the surface seawater of Jiaozhou Bay， the average mass concentration of perfluorooctanoic acid （PFOA） has significantly decreased. Additionally， potassium 9-chlorohexadecafluoro-3-oxanonane-1-sulfonate （F-53B）， one of the newly emerging PFASs， was detected in the surface seawater of Jiaozhou Bay， which may be attributed to the recent shift in PFASs production. The widespread use of these new PFASs may introduce environmental risks. These risks resemble those of traditional PFASs and require urgent attention. In summary， this method is operationally straightforward， rapid， and highly sensitive； it is suitable for analyzing the eight PFASs in seawater. This approach can provide a valuable data foundation and scientific basis for research and analysis concerning PFASs in environmental water bodies.

全氟及多氟烷基化合物（per- and polyfluoroalkyl substances， PFASs）是一类高度稳定的持久性有机新污染物，因其优异的表面活性和化学稳定性^［1，2］^，在纺织、造纸和消防等多个行业得到了广泛的应用^［3］^。然而，随着PFASs的大量使用，其对人体内分泌、免疫、生殖等系统的毒性和致癌风险引起了广泛关注^［4-6］^。我国GB 5749-2022《生活饮用水卫生标准》中规定全氟辛烷磺酸（perfluorooctane sulfonic acid， PFOS）和全氟辛酸（perfluorooctane carboxylic acid， PFOA）的检出质量浓度应分别低于40 ng/L和80 ng/L。近年来，传统长链全氟烷基化合物PFOA和PFOS的禁止生产使用促进了大量工业替代品如全氟-9-氯-3-氧杂壬烷磺酸钾（potassium 9-chlorohexadecafluoro-3-oxanonane-1-sulfonate， F-53B）、全氟-11-氯-3-氧杂十一烷磺酸（potassium 11-chloroeicosafluoro-3-oxaundecane-1-sulfonate， Minor F-53B）等新型PFASs的研发和应用^［7］^。然而，这些新型PFASs表现出与PFOS和PFOA相当的毒性，大量使用可能带来同样的环境风险^［8］^。因此，开发能够高效、灵敏检测水中PFASs的分析方法至关重要。

目前，固相萃取-超高效液相色谱-串联质谱（SPE-UHPLC-MS/MS）是检测PFASs的主要技术，但其在环境水体分析中存在样品前处理时间长的问题。分散膜萃取（dispersive membrane extraction， DME）作为一种新型的快速样品前处理技术，通过优化膜材料可提升萃取效率并加快传质。金属有机骨架（metal-organic frameworks， MOFs）是一类由有机配体与金属（簇）配位构建的多孔材料^［9］^，具有空隙结构可设计、比表面积大、传质快等优势^［10］^，目前已被用于样品前处理领域^［11，12］^。本课题组在前期工作中研制了高选择性阳离子型金属有机骨架复合膜材料（F-TMU-66^+^Cl^-^/PVDF MMM）^［13］^，其以氯化锆（ZrCl₄）、异烟酸氮氧化物（pyridine-4-carboxylic acid N-oxide， INO）及四氟对苯二甲酸（tetrafluoroterephthalic acid， F-H_2_BDC）为构筑单元，与聚偏二氟乙烯（poly vinylidene fluoride， PVDF）复合形成多孔膜。将基于材料开发的样品前处理技术与UHPLC-MS/MS结合成功建立了传统PFASs的检测方法，但目前尚未应用于海水分析。

胶州湾是位于山东半岛南部的典型半封闭海湾，其污染物易受陆源输入的影响。现有研究多关注入湾河流及排污口中PFASs的污染状况^［14，15］^，对胶州湾中PFASs及新型替代物（如F-53B）的赋存规律认知不足。

本研究聚焦于胶州湾海域PFASs的环境分布特征研究，基于分散膜固相萃取-超高效液相色谱-质谱联用技术，系统建立了适用于海水基质中PFASs的分析方法。在方法构建中重点优化了色谱分离条件（流动相中有机相类型、无机相中乙酸铵浓度）与质谱电离参数（离子源电压），显著提升了8种PFASs在复杂海水基质中的分离效率和响应信号。利用该方法在胶州湾表层海水中检出了新型多氟烷基化合物F-53B的存在，并结合水文数据解析了8种PFASs浓度梯度与陆源输入的关系。所建立的方法成功克服了痕量检测、基质复杂等对PFASs检测的影响，为近海PFASs污染溯源与生态风险评价提供了准确、高效且灵敏的分析支撑。

## 1 实验部分

### 1.1 仪器、试剂与材料

QTRAP 3500超高效液相色谱-三重四极杆质谱仪（美国AB Sciex公司），HA-C型水浴恒温振荡器（常州国宇仪器制造有限公司），ND200型氮气吹干仪（杭州瑞诚仪器有限公司），Millipore D-24UV超纯水机（美国Millipore公司）。

全氟丁酸（PFBA，纯度98%）和Minor F-53B（纯度99%）购自上海麦克林生化科技有限公司，PFOA（纯度96%）和全氟己烷磺酸（PFHxS，纯度95%）购自上海阿拉丁化学试剂有限公司，全氟壬酸（PFNA，纯度97%）购自上海阿法埃莎化学有限公司，PFOS（纯度96%）购自德国Dr. Ehrenstorfer公司，F-53B（纯度99%）购自加拿大惠灵顿实验室股份有限公司，全氟-2，5-二甲基-3，6-二氧杂壬酸（HFPO-TA，纯度95%）购自北京迈瑞达科技有限公司。以上8种PFASs的物理化学性质见表1。

**表1:** 8种PFASs的物理化学性质

Analyte	*M*_r_	p*K*_a_	log *K*_ow_^*^
Heptafluorobutyric acid （PFBA）	214.04	-0.11	2.04
Perfluorooctane carboxylic acid （PFOA）	414.07	0.50	0.50
Tridecafluorohexane1-sulfonic acid （PFHxS）	400.12	-3.34	3.93
Perfluorononanoic acid （PFNA）	464.08	0.52	4.46
2，5-Bis（trifluoromethyl）-3，6-dioxaundecafluorononanoyl fluoride （HFPO-TA）	496.07	6.43	0.42
Perfluorooctane sulfonic acid （PFOS）	500.13	-3.27	5.14
Potassium 9-chlorohexadecafluoro-3-oxanonane-1-sulfonate （F-53B）	554.17	/	6.04
potassium 11-chloroeicosafluoro-3-oxaundecane-1-sulfonate （Minor F-53B）	670.62	/	7.25

* Data were calculated using ChemDraw Professional v20.0； /： not available.

ZrCl_4_（纯度99.5%）购自上海麦克林生化科技有限公司，F-H_2_BDC（纯度97%）和INO（纯度99%）购自上海阿拉丁化学试剂有限公司；PVDF（纯度99%）购自美国阿克玛公司。丙酮（分析纯）、氨水（分析纯）和*N，N*-二甲基甲酰胺（分析纯）购自国药集团试剂化学有限公司。甲醇（色谱级）和乙腈（色谱级）购自德国默克公司。所有采集的水样经0.45 μm滤膜过滤后，装入聚丙烯材质的塑料瓶中，避光保存于4 ℃冰箱中。

### 1.2 样品采集与保存

按照我国国家标准《海洋调查规范第 4 部分：海水化学要素调查》（GB/T 12763.4-2007），于2023年10月在胶州湾海域设置11个采样站点，共采集11个表层海水样本（每个站点采集1个水样）。采样站点的位置如图1所示。海水样品均由采水器于0.5 m水深处采集。将采集的样品带回实验室后，于4 ℃暗处保存，并在样品采集后7 d内完成检测。

**图1:**
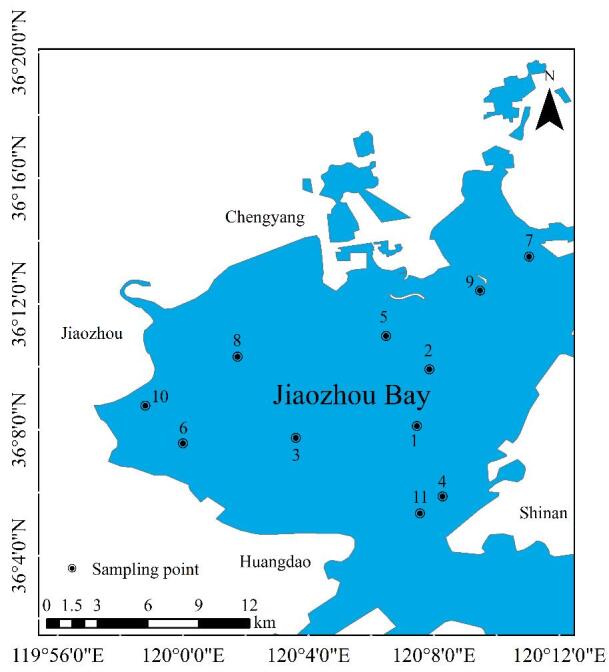
胶州湾采样点位

### 1.3 标准溶液的配制

分别称取8种PFASs标准品各4 mg，用乙腈溶解并定容至1 mL，配制质量浓度为4 g/L的标准储备液，置于棕色小瓶中，于4 ℃下储存。移取25 μL各标准储备液，用乙腈稀释定容至1 mL，配制质量浓度为100 mg/L的混合标准储备液。使用时，以乙腈为溶剂将混合标准储备液配制成质量浓度为0.1、0.5、1、5、25、35、50 μg/L的系列混合标准溶液。为得到方法曲线，将8种PFASs混合标准储备液用海水逐级稀释至质量浓度为0.5、0.8、1.5、2.0、5.0、10.0、50.0和100.0 ng/L的目标溶液。

### 1.4 样品前处理流程

采用本课题组前期工作中已优化的固相萃取参数对水样进行前处理^［13］^。F-TMU-66^+^中功能化有机配体F-H₂BDC与INO投加的物质的量比为3∶1，最优前处理流程及条件如下：首先将负载15 mg F-TMU-66^+^的混合基质膜（F-TMU-66^+^Cl^-^/PVDF MMM）浸入50 mL海水样品中（PFASs加标质量浓度100 ng/L），在30 ℃恒温振荡器中萃取25 min。完成萃取后取出F-TMU-66^+^Cl^-^/PVDF MMM转移至烧杯中，加入8 mL体积分数为7%的氨水甲醇溶液进行30 min振荡洗脱。洗脱液在40 ℃下氮吹浓缩至近干，用200 μL体积分数为40%的乙腈水溶液复溶，经0.22 μm滤膜过滤后采用UHPLC-MS/MS检测。

### 1.5 仪器条件

色谱柱：ACQUITY UPLC BEH C18 （100 mm×2.1 mm， 1.7 μm）；柱温：40 ℃；流速：0.4 mL/min；进样体积：10 μL；流动相：A相为1 mmol/L乙酸铵水溶液，B相为乙腈；洗脱梯度：0~12.0 min， 95%A~5%A； 12.0~15.0 min， 5%A； 15.0~15.1min， 5%A~95%A； 15.1~16.0 min， 95%A。

质谱离子源：负离子模式电喷雾离子（ESI）源；离子源电压：-2 500 V；离子源温度：300 ℃；多反应监测模式；8种PFASs的其他质谱参数见表2。

**表2:** 8种PFASs的质谱参数

Analyte	*t*_R_/min	Precursor ion （*m/z*）	Product ions （*m/z*）	Declustering potentials/V	Collision energies/eV
PFBA	3.20	212.8	168.7^*^	-39.1	-12.0
PFOA	6.02	413.1	368.6^*^，168.9	-52.7， -53.1	-15.8， -26.9
PFHxS	6.22	398.9	79.9^*^，99.0	-119.8， -106.0	-77.0， -74.0
PFNA	6.63	463.0	418.7^*^，218.9	-51.1， -26.9	-16.7， -23.8
HFPO-TA	6.93	495.2	185.0^*^，450.4	-24.0， -22.0	-9.2， -5.6
PFOS	7.84	498.9	79.6^*^，98.8	-140.0， -140.8	-98.2， -98.2
F-53B	8.14	530.8	350.7^*^，82.7	-107.7， -122.0	-36.0， -70.0
Minor F-53B	9.88	630.7	450.9^*^	-113.0	-39.9

* Quantitative ion.

## 2 结果与讨论

### 2.1 液相色谱条件优化

#### 2.1.1 流动相中有机相的选择

对比了1.0 mmol/L乙酸铵水溶液-甲醇和1.0 mmol/L乙酸铵水溶液-乙腈两种流动相体系对8种PFASs峰形特征和响应情况的影响。结果（见图2）显示，采用乙腈作为流动相时，目标物分离效果更好，峰信号响应强度更高。此外，在ESI过程中，乙腈由于其高挥发性和低表面张力的特点，更容易形成微小液滴，这种特性有利于提高离子化效率。因此，选择1.0 mmol/L乙酸铵水溶液与乙腈作为流动相。

**图2:**
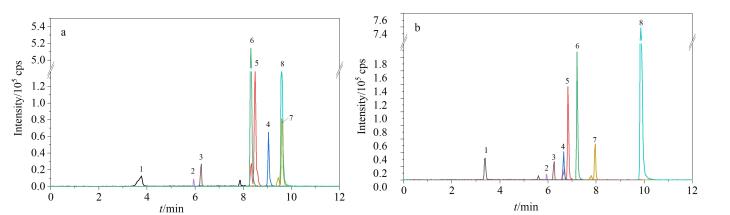
使用（a）甲醇和（b）乙腈作为有机流动相时PFASs的色谱图

#### 2.1.2 流动相中水相乙酸铵浓度的选择

流动相pH值直接影响目标物在反相色谱中的离子形态和在色谱柱中的保留特性。本研究拟通过在流动相中加入一定浓度的pH调节剂以改善目标物的离子化效率及色谱保留行为。乙酸铵是具有缓冲能力的盐类，可增加离子化效率，具有溶解性好、对色谱柱伤害小、不易引入背景噪声等优点，常用于PFASs的液相色谱-质谱法分析中^［16］^。本文对乙酸铵的浓度进行了优化，并考察了不同乙酸铵浓度（0.1、0.5、1.0、2.0、5.0 mmol/L）对8种PFASs响应程度的影响。结果如图3所示，随着乙酸铵浓度的升高，标准溶液中8种PFASs的响应强度呈现先增后降的趋势。当乙酸铵浓度为1.0 mmol/L时，响应强度达到最佳。当乙酸铵浓度超过1.0 mmol/L时，响应强度开始下降，这可能是由于高浓度乙酸铵会引起离子抑制效应。因此，本研究最终确定乙酸铵的浓度为1.0 mmol/L。

**图3:**
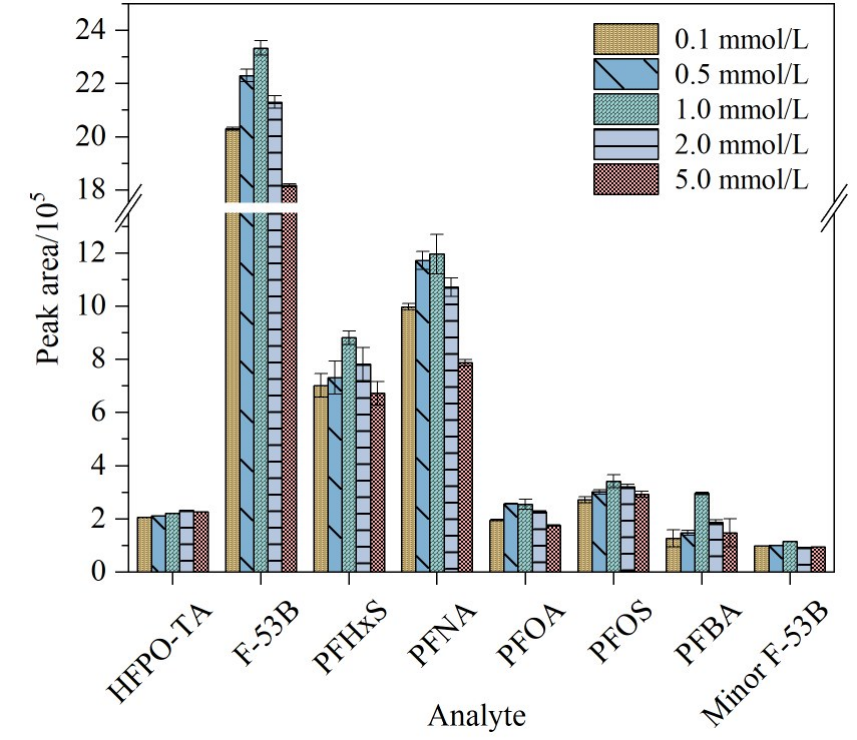
水相中加入不同浓度乙酸铵对8种PFASs峰面积的影响（*n*=3）

### 2.2 质谱条件优化

离子源电压作为质谱分析的关键控制参数，通过调节ESI过程中的电荷转移效率，直接影响分析物的离子化效能。在ESI电离机制中，施加于金属喷针的高电压诱导溶液带电，形成带电液滴。随着溶剂的持续挥发与库仑分裂作用，液滴表面电荷密度逐步增大，电荷逐渐转移到分析物分子（如PFASs）上，最终生成气相带电离子。这些离子在电场作用下经离子传输管进入质量分析器，最终被检测器捕获^［16］^。因此，离子源电压对样品中的目标分子电离效率有直接影响，进而对UHPLC-MS/MS检测8种PFASs的信号强弱起关键作用。本实验考察了不同离子源电压（-2 500、-3 500和-4 500 V）对信号强度的影响。结果如图4所示，同一化合物在不同电压下峰面积变化不大。总体而言，大部分目标化合物的响应强度在离子源电压为-2 500 V时达到最大值，而较高的电压则导致信号强度下降。这一现象可能与高电压引起的被测物分解有关，过多的离子进入质谱后，与溶剂或自身离子发生碰撞并产生裂解。因此，本实验最终将离子源电压设定为-2 500 V。

**图4:**
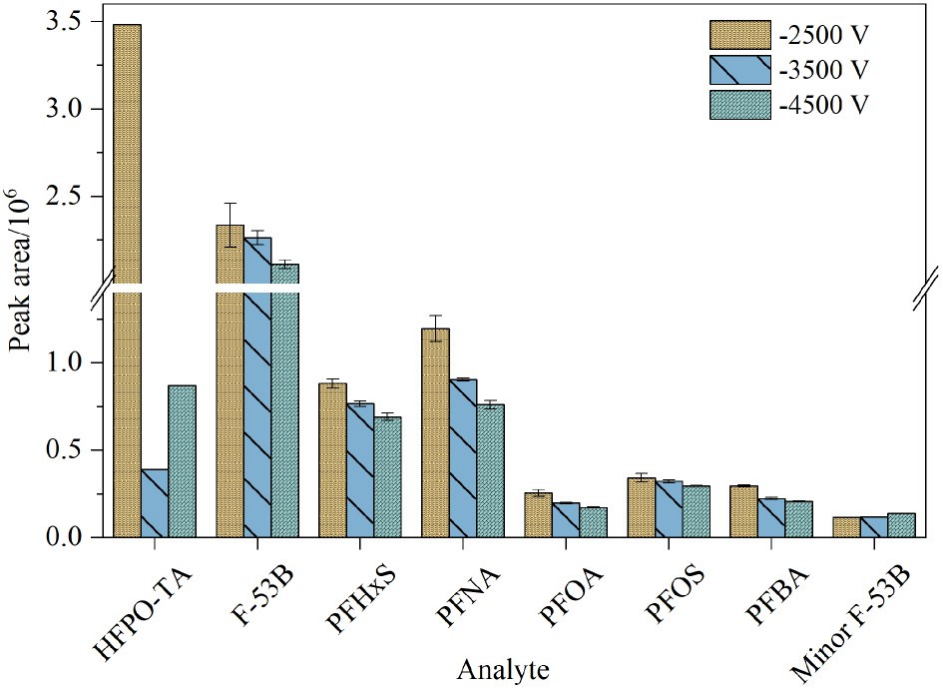
不同离子源电压对8种PFASs峰面积的影响（*n*=3）

### 2.3 方法学验证

由于PFASs用途广泛且易在玻璃装置和含氟制品上残留，实验中选择聚乙烯或聚丙烯材质的器皿。为确保数据的准确性，在样品检测的过程中同步进行试剂空白对照实验，并对所有数据进行空白值扣除处理。在最优条件下对8种PFASs进行线性和灵敏度考察，结果如表3所示，PFOA、PFOS和F-53B在0.5~100.0 ng/L范围内，PFNA和Minor F-53B在0.8~100.0 ng/L范围内，PFBA、PFHxS和HFPO-TA在2.0~100.0 ng/L范围内均呈现良好的线性关系，线性相关系数（*r* ^2^）为0.990 7~0.999 6。方法的检出限（LOD）和定量限（LOQ）分别为0.07~0.49 ng/L和0.22~1.63 ng/L，该方法的LOQ低于我国GB 5749-2022《生活饮用水卫生标准》中对PFOS（40 ng/L）和PFOA（80 ng/L）的规定限值，满足定量分析的基本要求。

**表3:** 8种PFASs的分析方法性能参数

Analyte	Linear range/（ng/L）	Linear equation	*r* ^2^	LOD/（ng/L）	LOQ/（ng/L）
Minor F-53B	0.8-100	*y*=81.96*x*+599.20	0.9907	0.19	0.64
F-53B	0.5-100	*y*=7608.50*x*-22923.00	0.9952	0.07	0.22
PFOS	0.5-100	*y*=2403.10*x*-2992.30	0.9972	0.09	0.28
PFNA	0.8-100	*y*=1988.20*x*-5861.30	0.9974	0.20	0.65
PFHxS	2.0-100	*y*=773.62*x*+2489.40	0.9996	0.49	1.63
PFOA	0.5-100	*y*=1367.40*x*+5714.70	0.9960	0.09	0.31
PFBA	2.0-100	*y*=137.69*x*+341.71	0.9991	0.47	1.56
HFPO-TA	2.0-100	*y*=47.87*x*+193.67	0.9947	0.48	1.59

*y*： peak area； *x*： mass concentration， ng/L.

此外，在空白海水基质中分别进行3个浓度梯度的加标实验，通过添加PFASs混合标准溶液使最终海水样品中目标物的质量浓度分别达到10.0、50.0和100.0 ng/L，按照上述前处理方法进行加标回收试验，计算加标回收率和相对标准偏差（RSD）。结果表明，8种PFASs的加标回收率为50.4%~116.4%，日内和日间的RSD分别为1.0%~19.2%和2.2%~19.5%，结果见表4。该结果表明此方法的重复性和准确性良好，适用于海水样品中8种PFASs的测定。

**表4:** 8种PFASs的加标回收率及相对标准偏差（*n*=3）

Analyte	Spiked/ （ng/L）	Recovery/%	Intra-day RSD/%	Inter-day RSD/%
PFBA	10	100.1	19.2	18.0
	50	102.7	9.1	2.2
	100	115.8	2.5	7.7
HFPO-TA	10	116.4	18.9	15.3
	50	86.3	17.8	18.2
	100	71.3	12.1	15.5
PFOA	10	116.1	9.4	14.8
	50	62.8	3.9	5.2
	100	50.4	15.3	18.2
PFHxS	10	94.9	14.6	14.8
	50	108.2	16.7	10.6
	100	97.5	4.7	8.1
PFNA	10	103.0	14.6	17.9
	50	64.8	16.9	14.8
	100	63.0	1.0	16.8
PFOS	10	103.6	4.6	4.5
	50	91.9	13.8	11.7
	100	77.1	13.4	11.3
F-53B	10	88.8	4.8	14.0
	50	80.7	12.9	19.5
	100	83.4	4.7	17.8
Minor F-53B	10	71.1	11.0	15.4
	50	108.2	6.4	14.7
	100	76.1	8.0	17.9

采用我国GB/T5750.8-2023《生活饮用水标准检验方法第8部分：有机物指标》中的方法进行样品前处理实验。将标准方法中基于弱阴离子交换（WAX）固相萃取柱建立的SPE-UHPLC-MS/MS方法和本研究中基于阳离子型金属有机骨架膜材料F-TMU-66^+^Cl^-^/PVDF MMM建立的DME-UHPLC-MS/MS方法同时用于海水样品中PFASs的测定，两种方法测定PFASs代表物PFNA的平均质量浓度分别为20.02 ng/L和19.07 ng/L，在*n*=5、置信度为95%时，计算得到的*t*值为1.84。查表得*t*_0.05（4）_值为2.78，且*t*_0.05（4）_>*t*_计算_，说明使用两种方法在测定结果上无显著性差异，然而本方法与国标方法相比，具有操作简单，无需购买特殊设备的优势。

因此，基于F-TMU-66^+^Cl^-^/PVDF MMM的DME-UHPLC-MS/MS分析方法与国标方法具有可比性，适用于海水中PFASs的准确高效分析。

### 2.4 实际水样分析

实验结果显示，在胶州湾采集的11个表层海水样品中共检测出7种PFASs（PFHxS未检出），其质量浓度范围为19.47~99.51 ng/L（平均值54.96 ng/L，中位值55.01 ng/L），各组分质量浓度中位值由高到低依次为：Minor F-53B（12.71 ng/L）>PFBA（10.21 ng/L）>HFPO-TA（8.03 ng/L）>PFOA（6.21 ng/L）>PFNA（4.25 ng/L）>PFOS（3.69 ng/L）>F-53B（3.4 ng/L）。各组分的质量浓度和组成占比如图5所示。新型及短链PFASs（Minor F-53B、PFBA、HFPO-TA）在∑PFASs中的总占比达到73.8%，显著高于长链PFASs。与2018年同区域监测数据（PFOA平均质量浓度11.58 ng/L）^［17］^相比，本研究中PFOA平均质量浓度降至5.53 ng/L，表明该污染物浓度呈下降趋势。值得注意的是，作为PFOA的工业替代品，含氟聚合物生产过程中使用的HFPO-TA在本研究中检出（8.99 ng/L）。此外，本研究在胶州湾海域检出PFOS替代物F-53B（3.57 ng/L）。该物质作为中国电镀行业广泛使用的新型PFASs，其生产应用量近年来呈现持续增长态势，并已在多种环境介质中被检出^［18］^。2022年，中国生态环境部发布《重点管控新污染物清单（2023年版）》，将PFOA及其衍生物、PFOS类物质列为优先管控对象^［19］^。监测数据显示，胶州湾传统PFASs浓度呈现下降趋势，政策实施与污染物减排趋势高度吻合，表明中国政府对传统PFASs的管控政策已初见成效。

**图5:**
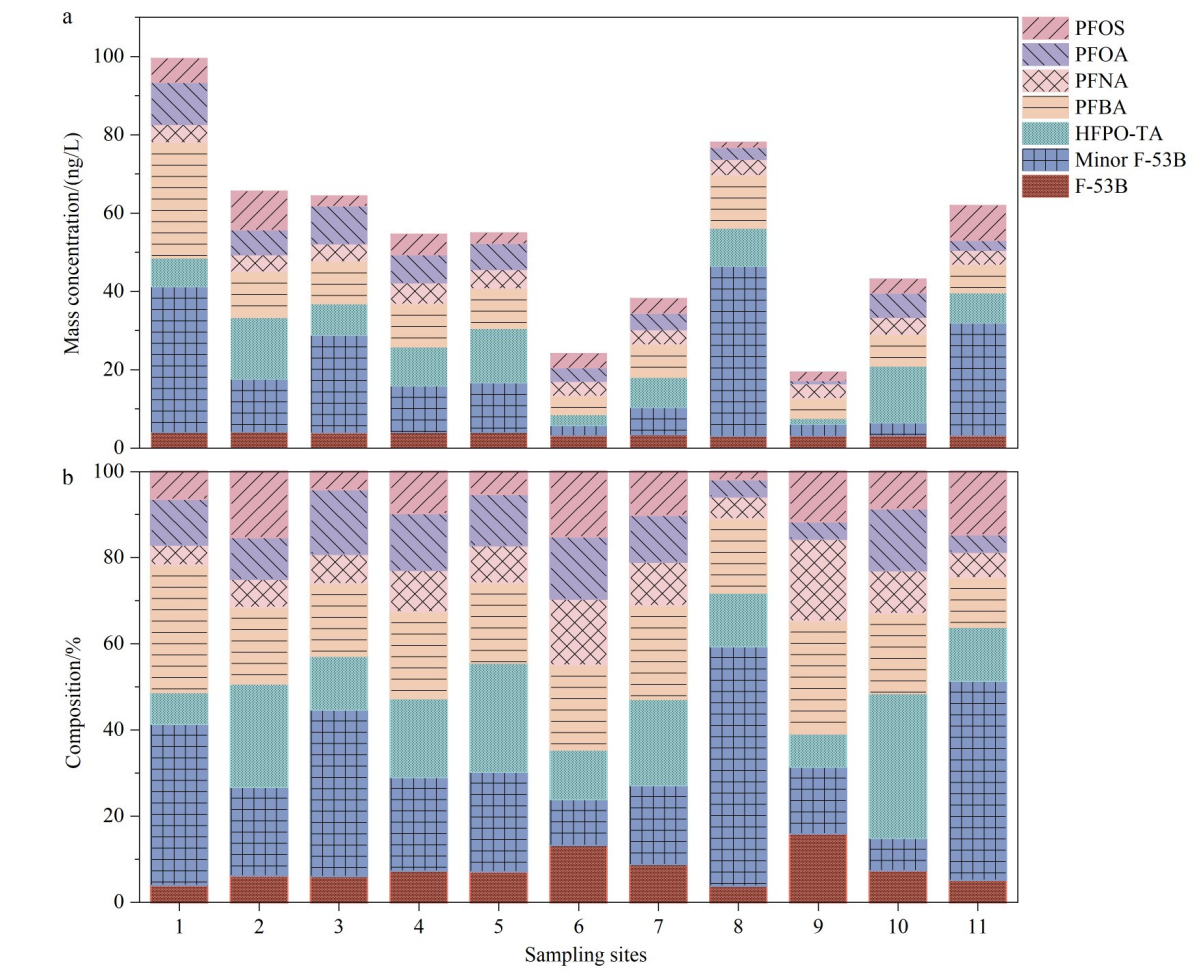
胶州湾表层海水样品中PFASs的（a）质量浓度水平和（b）组成占比

据文献报道，2019年在长江支流的沱江中共检出7种PFASs，其中PFOA占主导地位（贡献率为41.4%，984.00 ng/L），F-53B检出率达100%，质量浓度为0.39 ng/L^［20］^。2020年渤海湾共检测出12种PFASs，其中PFOA占主导地位（贡献率为32%，质量浓度14.96 ng/L），F-53B检出质量浓度较低，为0.28 ng/L，贡献率为0.6%^［21］^。2021年，珠江口处共检出17种PFASs，其中PFOA占主导地位（贡献率为41.4%，质量浓度20.90 ng/L）^［22］^。与其他海域中PFASs的检测结果相比，胶州湾污染物分布与渤海湾和珠江口略有不同，Minor F-53B质量浓度占比较大，说明该区域电镀行业对PFOS替代品使用较多。

为初步探究胶州湾表层海水中PFASs的空间分布特征，本研究选取7种检出率均为100%的PFASs作为检测对象。其空间分布呈现显著差异：1号点位表层海水中∑PFASs质量浓度最高（99.51 ng/L），其次为8号点位（78.14 ng/L），而9号点位质量浓度最低（19.47 ng/L）。空间分布差异可能与流域污染源排放强度有关：（1） 1号点位位于海泊河河口附近。海泊河作为青岛市主要的工业废水和生活污水纳污河道，其流域内分布着化工、造纸、印染、电镀、机械和纺织等密集的工业产业。河口处建有海泊河污水处理厂，主要处理周边城市的生活污水和工业废水，达标排放的废水通过直排口进入胶州湾。该流域污水排放总量在青岛市总排放量中占据较大比重^［17］^，污染负荷对近岸海域环境影响较大。（2） 8号点位毗邻大沽河入湾排污口，所在流域是青岛市化学品制造产业的核心聚集区，其氟化物生产活动可能持续释放以氟化物为原料的工业型PFASs污染物。流域内分布的249.4万常住人口产生的生活污水系统，可能将含PFASs的日用化学品（如洗涤剂、化妆品等）随污水输入湾体。工业排放与生活源排放的叠加效应，显著增加了该区域PFASs的污染通量^［14］^。（3） 9号点位低浓度特征可能与该区域附近的含氟化工企业的空间迁移（如向董家口经济区等区域转移）相关，显著减少了直接排入胶州湾的工业污染源。

## 3 结论

本文选用课题组前期开发的一种双功能型材料F-TMU-66^+^Cl^-^/PVDF MMM作为分散膜萃取中的萃取材料，并直接采用了前期研究所报道的优化后前处理条件进行后续实验。在色谱和质谱条件优化后，本文建立了一种基于DME结合UHPLC-MS/MS检测环境水样中8种PFASs的分析方法。通过*t*检验表明，该分析方法中的前处理技术与国家标准中的方法无显著性差异。本方法具有良好的灵敏度、精密度和准确度，可满足实际样品的检测要求。与往年胶州湾海水样品中PFASs的检测结果相比，本次检测发现随着传统PFASs的使用受限，新型PFASs的检出含量有所增加，说明存在工业转型的趋势。其大量使用可能导致与传统PFASs类似的环境风险，需引起重视。本工作不仅为海水中PFASs的检测提供了方法支撑，还可为水体环境中传统与新型PFASs的污染监测及潜在风险预警等提供科学数据支撑与理论依据。
